# The combination effect of meropenem/sulbactam/polymyxin-B on the pharmacodynamic parameters for mutant selection windows against carbapenem-resistant *Acinetobacter baumannii*

**DOI:** 10.3389/fmicb.2022.1024702

**Published:** 2022-11-22

**Authors:** Jiayuan Zhang, Shuo Diao, Yanfei Liu, Hongxiang Wang, Yuwei Liu, Shixing Zhu, Kun Feng, Xiaoqian Tang, Charles Oo, Peijuan Zhu, Zhihua Lv, Mingming Yu, Sherwin K. B. Sy, Yuanqi Zhu

**Affiliations:** ^1^School of Medicine and Pharmacy, Ocean University of China, Qingdao, China; ^2^Department of Laboratory Medicine, The Affiliated Hospital of Qingdao University, Qingdao, China; ^3^Laboratory of Pathology and Immunology of Aquatic Animals, KLMME, Ocean University of China, Qingdao, China; ^4^SunLife Biopharma, Morris Plains, NJ, United States; ^5^Department of Pharmacology, University of Pennsylvania, Philadelphia, PA, United States; ^6^Laboratory for Marine Drugs and Bioproducts of Qingdao National Laboratory for Marine Science and Technology, Qingdao, China; ^7^Department of Statistics, State University of Maringá, Maringá, Brazil

**Keywords:** *Acinetobacter baumannii*, OXA-23, polymyxin-B, meropenem, sulbactam, pharmacodynamics

## Abstract

The objective of this study was to evaluate whether combinations of sulbactam, meropenem, and polymyxin-B could reduce or close the gap of mutant selection window (MSW) of individual antibiotics against *Acinetobacter baumannii* harboring OXA-23. MICs of three antimicrobials used alone and in combination (meropenem/polymyxin-B or meropenem/polymyxin-B/sulbactam) were obtained in 11 clinical isolates and mutant prevention concentrations were determined in 4 of the 11 isolates. All isolates were resistant to meropenem or polymyxin-B. Combining meropenem and polymyxin-B with or without sulbactam resulted in synergistic bactericidal activities. Pharmacokinetic (PK) simulations of drug concentrations in the blood and epithelial lining fluid coupled with pharmacodynamic (PD) evaluations revealed that the fractions of time over the 24-h in terms of free drug concentration within the MSW (*f*T_MSW_) and above the MPC (*f*T_>MPC_) were optimized by combination therapy. The resultant clinical regimens of meropenem, polymyxin-B, and sulbactam evaluated in the PK-PD analysis were 2 g q8h, 2.5 mg/kg loading dose followed by 1.5 mg/kg q12h, and 3 g q8h, respectively, in patients with normal renal function. Subsequent corresponding equivalent exposure regimens would depend on the extent of renal failure. The overall results indicate that combination antibiotics consisting of sulbactam/meropenem/polymyxin-B can confer potential efficacy against *A. baumannii* harboring OXA-23, and reduce the opportunity for bacteria to develop further resistance. This study provides a framework for pharmacodynamic evaluation of drug-resistant mutant suppression in an antimicrobial co-administration setting. The results thereby lay the groundwork for additional studies and future clinical confirmation is warranted.

## Introduction

*Acinetobacter baumannii* has emerged as a serious pathogen causing nosocomial infections, including meningitis, hospital-acquired and ventilator-associated pneumonia (HAP & VAP), line-associated bloodstream infection, catheter-associated urinary tract infections, and skin and soft tissue infections (Maragakis and Perl, 2008). Infections due to carbapenem-resistant *A. baumannii* (CRAB) are associated with high morbidity and mortality rates which can be as high as 60% among intensive care unit patients (Wong et al., 2017). The CRAB can resist major antimicrobials (penicillins, aminoglycosides, expanded-spectrum cephalosporins, and fluoroquinolones) and survive in healthcare facilities, posing tremendous challenges for healthcare providers (Peleg et al., 2008; Manchanda et al., 2010). Carbapenems are the drugs of choice against *Acinetobacter* infections but are currently being compromised by the emergence of class B and D β-lactamases in these pathogens (Livermore, 2002). However, recently approved β-lactamase inhibitors, including avibactam and vaborbactam, have minimum activities against *A. baumannii* (Pogue et al., 2019). Consequently, CRAB was categorized as an urgent level threat by the Centers for Disease Control and Prevention (Papp-Wallace et al., 2011; Meletis, 2016).

Carbapenem resistance in *A. baumannii* is acquired by several proposed mechanisms: the presence of β-lactamases (class B metallo-β-lactamases – MBL, or class D oxacillinases – OXA), the loss of outer membrane porins, overexpression of efflux pumps and alterations in penicillin-binding proteins (Nguyen and Joshi, 2021). In terms of genomics, the *bla*_OXA-23_ gene is prevalent in CRAB (Mugnier et al., 2010), and its expression confers resistance to both meropenem and sulbactam(Sgrignani et al., 2016; Yang et al., 2019). Oxacillinases in CRAB are chromosomal enzymes that can be intrinsic (e.g., OXA-51-like) or acquired, as in the case of OXA-23-like expression (Kusradze et al., 2011). Notably, outbreaks of OXA-23-producing CRAB have been reported worldwide (Dalla-Costa et al., 2003; Mugnier et al., 2010; Zhao et al., 2019).

The resurgence of polymyxin utilization in the clinic is contributing to polymyxin resistance in *A. baumannii*. Moreover, *A. baumannii* with mixed susceptibility patterns or heteroresistance is difficult to be detected using standard susceptibility testing methods (Yau et al., 2009; Barin et al., 2013). This rapid adaptive resistance to polymyxin-B in OXA-23-producing CRAB isolates could complicate treatments using polymyxins (Barin et al., 2013). Therefore, combination antibiotic therapy with optimized dosage regimens has been proposed, essentially as a way to limit emerging resistance and increase antimicrobial activities in patients treated with polymyxins (Bergen et al., 2015).

To showcase synergistic activities of sulbactam/meropenem/polymyxin-B combination against OXA-23-producing CRAB isolates, *in vitro* studies utilizing a hollow-fiber infection model and susceptibility evaluation were carried out (Lenhard et al., 2017; Menegucci et al., 2019). Anecdotal clinical experiences suggested reliable translatability of *in vitro* synergistic activities to clinical treatment against colistin-resistant *A. baumannii* (Qureshi et al., 2015). In this study, we investigated the effects of a two-antibiotic combination of meropenem and polymyxin-B and a triple-antibiotic combination of sulbactam, meropenem, and polymyxin-B on relevant pharmacodynamic parameters in CRAB harboring OXA-23 mutant.

## Materials and methods

### Antimicrobial agents

Analytical-grade amikacin, amoxicillin, aztreonam, clindamycin, colistin, polymyxin-B, meropenem, rifampicin, sulbactam, and vancomycin were obtained from the Shanghai Macklin Biochemical Co. Ltd. (Shanghai, China). Stock solutions containing each antibiotic were separately prepared at a concentration of 10.24 mg/l according to the Clinical Laboratory and Standards Institute guidelines (CLSI, 2020).

### Bacterial isolates

Clinical isolates of *A. baumannii* were collected from the oral mucus of various pneumonia patients admitted to the Affiliated Hospital of Qingdao University between 2019 to 2021 (Ethics committee approval no.: QDFY20180512). The clinical isolates were provided by Dr. Yuanqi Zhu. Next-generation sequencing was used to determine the resistance genes in each isolate, as described (Feng et al., 2021). Eleven clinical *A. baumannii* strains harboring *bla*_OXA-23_ were selected. *E. coli* ATCC 25922 and *A. baumannii* ATCC 19606 were used as a quality control strain for antimicrobial susceptibility tests.

### Susceptibility testing

Susceptibility of each clinical isolate to amikacin, amoxicillin, aztreonam, clindamycin, colistin, rifampicin, and vancomycin as monotherapy was evaluated by broth microdilution. A checkerboard assay was used to determine the susceptibility profile of each clinical isolate to the three antibiotics (meropenem, polymyxin-B, and sulbactam) either used alone or in combination with each determination conducted in triplicate. The concentration ranges of polymyxin-B and meropenem alone and in combination were 1 to 64 mg/l and 1 to 128 mg/l, respectively, whereas sulbactam was fixed at 4 mg/l for the triple-antibiotic combination, since ampicillin/sulbactam susceptible breakpoint is ≤8/4 mg/l (CLSI, 2020). Setting sulbactam concentration at the susceptible breakpoint ensures that the clinical dosing regimen of ampicillin/sulbactam provides sufficient activity at this MIC.

Based on the results of the checkerboard assay, the fractional inhibitory concentration index (FICI) was calculated


$$\begin{array}{c} \mathrm{FICI}=\frac{MIC\;ofantibiotic1\;incombination}{MIC\;ofantibiotic1\;alone}+\\ \frac{MIC\;ofantibiotic2\;incombination}{MIC\;ofantibiotic2\;alone}+\\ \frac{MIC\;ofantibiotic3\;incombination}{MIC\;ofantibiotic3\;alone} \end{array}$$

When FICI is ≤0.5, the two drugs are considered synergistic; FICI >0.5–1 is additive; >1- < 2 indicates indifference; and ≥ 2 is antagonistic (Hall et al., 1983).

A final high-density inoculum of ≥10^10^ cfu/ml was used to determine the mutant prevention concentration (MPC) of four selected isolates to polymyxin-B and meropenem used alone and in combination. The four isolates were selected such that meropenem MIC values in combination with polymyxin-B and sulbactam were below the resistant breakpoint. A high inoculum size ensured the emergence of the first-step mutants (Dong et al., 2000). A series of Mueller-Hinton agar plates containing the concentration of antimicrobial agent alone or in combination at 1×, 2×, 4×, 8×, 16×, and 32 × MIC were plated with approximately 100 μl of the high-density inoculum. Sulbactam concentration was fixed at 4 mg/l in the triple-antibiotic combination. The MPC was determined to be the lowest antimicrobial concentration that completely prevented bacterial growth after 72 h incubation at 35 ± 2°C. The mutant selection window (MSW) is defined as the concentration range between MIC and MPC.

### Time–kill kinetics

Time-kill kinetic studies were performed to evaluate the *in vitro* time-course of bacterial response to meropenem, polymyxin-B, and sulbactam alone or in combination against two *A. baumannii* isolates (2 and E). The experiment had five groups: control, meropenem, polymyxin-B, meropenem/polymyxin-B, and meropenem/polymyxin-B/sulbactam. Two concentrations of meropenem and polymyxin-B were tested at MIC and 2 × MIC, whereas sulbactam concentration was fixed at 4 mg/l. The constant concentration time-kill studies were conducted in *A. baumannii* isolates 2 and E, as previously described (Sy et al., 2016, 2017). For isolate 2, the drug concentrations at MIC evaluated were: 16 mg/l meropenem, 16 mg/l polymyxin-B, 4 mg/l meropenem, and 4 mg/l polymyxin-B with and without 4 mg/l sulbactam. For strain E, the concentration at MIC evaluated was 128 mg/l meropenem, 16 mg/l polymyxin-B, 4 mg/l meropenem, and 1 mg/l polymyxin-B with and without 4 mg/l sulbactam. At 2 × MIC, the drug concentrations for meropenem and polymyxin-B were doubled except for sulbactam which was fixed at 4 mg/l.

### Pharmacokinetic simulations and pharmacodynamic indices in plasma and epithelial lining fluid

The demographical characteristics of the virtual patient population were simulated as previously described (Sy et al., 2014; Zhu et al., 2022a). Albumin was assumed to be normally distributed, 2.0 ± 0.5 g/dl (mean ± SD).

The intravenous dosing regimens for sulbactam, meropenem, and polymyxin-B are listed in Table 1. A Monte Carlo simulation of 10,000 drug concentration-time courses over a day was generated for each dosing regimen by renal function category. Reported population PK models were described in the Supplementary materials.

**Table 1:** Dosing regimens of meropenem/sulbactam/polymyxin-B used in simulation by their creatinine clearance category.

**Creatinine clearance**	**Dosing regimens**
	*meropenem / sulbactam*
>70 to 150 ml/min	2 g q8h / 3 g q8h as a continuous infusion
>50 to 70 ml/min	2 g q8h / 3 g q8h as 3 h infusion
>25 to 50 ml/min	2 g q12h / 3.5 g q12h as 4 h infusion
15 to 25 ml/min	1 g q12h / 1.5 g q12h as 4 h infusion
	*polymyxin-B*
All renal function	Loading dose 2.5 mg/kg followed by 1.5 mg/kg q12h at 12 h as 1 h infusion
All renal function	Loading dose 2.0 mg/kg followed by 1.25 mg/kg q12h at 12 h as 1 h infusion

The simulations of free drug concentration in the ELF assumed a fixed proportional ratio between ELF and plasma drug concentrations. ELF/plasma penetration ratios of 30% for meropenem and 52% for sulbactam came from human PK studies, whereas a 60% penetration ratio for polymyxin-B was derived from a mouse infection model (Lodise et al., 2011; He et al., 2013; Rodvold et al., 2018). The plasma protein binding of sulbactam and meropenem were 32 and 2%, respectively (Martins et al., 2020). Polymyxin-B plasma protein binding was highly variable, ranging from 50 to 92% (Zavascki et al., 2008; Sandri et al., 2013; Abodakpi et al., 2015). In the simulation, we assumed a fixed 80% protein binding in the plasma. Polymyxin-B is known to bind to surfactants in the lung such as mucus (Huang et al., 2015; Samad et al., 2019). Unbound polymyxin-B in the presence of mucin was 15%, which was used in the simulation.

The PD indices of meropenem and sulbactam were based on 50% *f*T_>MIC_ and 60% *f*T_>MIC_, respectively (Li et al., 2007; Yokoyama et al., 2014, 2015). In the case of sulbactam, a fixed MIC of 4 mg/l was assumed. For polymyxin-B, the PD index, area under the free concentration-time curve divided by MIC (*f*AUC/MIC), required for 1-log kill was at least 8.2 in the lung infection model (Bergen et al., 2012). These values were used in the determination of the probability of target attainment (PTA).

### Pharmacodynamic parameters for suppression of emergence of resistant mutant

The two PD parameters associated with the selection of resistant mutant were (1) fraction of time over the 24-h in terms of free drug concentration that was within MSW (*f*T_MSW_) and (2) fraction of time over the 24-h period in terms of free drug concentration that exceeded the MPC (*f*T_>MPC_). The *f*T_MSW_ was computed from the difference between *f*T_>MPC_ and *f*T_>MIC_ for both meropenem and polymyxin-B. Summary statistics of these two parameters were reported for select isolates.

### Software

Both PK simulations and PD evaluations were performed using the RxODE package and user-defined functions in R (version 4.1.2).

## Results

### *In vitro* antimicrobial susceptibility

All eleven *A. baumannii* isolates were resistant to various antibiotics including β-lactams, aminoglycosides, glycopeptides and other classes of antibiotics (Table 2). These isolates carried OXA-23 along with other β-lactamase genes and exhibited resistance to polymyxin-B and meropenem as well (Table 3). The MIC of meropenem alone against these isolates ranged from 16 to >128 mg/l, whereas the MIC of polymyxin-B alone ranged from 4 to 16 mg/l. MIC of sulbactam alone were ≥ 64 mg/l. CLSI breakpoints were used for the interpretation of polymyxin-B MIC results: ≤2 mg/l (intermediate), >2 mg/l (resistant); and meropenem MIC results: ≤ 2 mg/l (susceptible), 4 mg/l (intermediate), and ≥ 8 mg/l (resistant) for *A. baumannii* (CLSI, 2020).

**Table 2:** Minimum inhibitory concentrations of various antibiotics as monotherapy against clinical isolates of *A. baumannii*, showing resistance to common antibiotics.

*A. baumannii* clinical isolates	Minimum inhibitory concentration (mg/L)						
	Amikacin	Colistin	Rifampicin	Amoxicillin	Aztreonam	Clindamycin	Vancomycin
A	>128	16	4	>128	>128	>128	>128
C	>128	32	2	64	>128	>128	>128
E	>128	8	16	>128	64	>128	>128
F	>128	8	4	>128	>128	>128	>128
G	>128	32	4	>128	>128	>128	>128
2	16	16	8	>128	>128	128	>128
12	>128	16	4	>128	64	>128	>128
13	>128	16	4	>128	128	>128	>128
20	>128	8	4	>128	>128	>128	>128
21	>128	8	8	>128	>128	>128	>128
22	32	8	>64	>128	>128	>128	>128

**Table 3:** Minimum inhibitory concentrations of meropenem and polymyxin-B alone or in combination with or without sulbactam (fixed at 4 mg/l) against carbapenem-resistant *A. baumannii* isolates and fractional inhibitory concentration index. β-lactamase encoding genes of the clinical isolates are also listed.

Clinical isolates	β-lactamase encoding genes	Minimum Inhibitory Concentration (mg/L)							Synergism analysis	
		Monotherapy			Combination therapy					
		meropenem	polymyxin-B	sulbactam	meropenem/ sulbactam	meropenem/ polymyxin-B	polymyxin-B/ sulbactam	meropenem/ polymyxin-B/ sulbactam	FICI index^‡^	FICI category
*E. coli* ATCC25922		1	1	32	–	–	–	–	–	–
*A. baumannii*										
*ATCC19606*		≤1	2	64	–	–	–	–	–	–
A	ADC-25, OXA-23, OXA-66, TEM-1D	>128	8	>64	>128/4	≤1/2	8/4	≤1/2/4	0.3203	Synergy
C	ADC-25, OXA-23, OXA-66, TEM-1D	64	8	>64	64/4	8/8	8/4	8/2/4	0.4375	Synergy
E	ADC-25, OXA-23, OXA-66, TEM-1D	>128	16	>64	>128/4	4/≤1	4/4	4/≤1/4	0.1563	Synergy
F	ADC-25, OXA-23, OXA-66, TEM-1D	>128	8	>64	>128/4	8/2	8/4	2/2/4	0.3281	Synergy
G	ADC-25, OXA-23, OXA-66, TEM-1D	>128	4	>64	>128/4	4/2	4/4	≤1/2/4	0.5703	Indifferent
2	ADC-25, OXA-23, OXA-66, TEM-1D	16	16	>64	16/4	4/4	8/4	4/4/4	0.5625	Indifferent
12	ADC-25, OXA-23, OXA-66, TEM-1D	64	16	>64	64/4	8/2	8/4	8/2/4	0.3125	Synergy
13	ADC-25, OXA-23, OXA-66, TEM-1D	64	16	>64	64/4	8/2	8/4	2/2/4	0.2188	Synergy
20	ADC-25, OXA-23, OXA-66	32	8	>64	32/4	2/2	8/4	≤1/2/4	0.3438	Synergy
21	ADC-25, OXA-23, OXA-66	64	16	>64	64/4	8/2	8/4	8/2/4	0.3125	Synergy
22	ADC-25, OXA-23, OXA-66, TEM-1D	32	8	>64	32/4	16/8	8/4	16/2/4	0.8125	Indifferent
MIC50		64	8	>64	64/4	8/2	8/4	4/2/4		
MIC90		>128	16	>64	>128/4	16/8	8/4	16/2/4		

MIC, minimum inhibitory concentration; FICI, fractional inhibitory concentration index.

‡FICI score was computed using the reduced MICs of meropenem, polymyxin-B and sulbactam in the triple combination relative to meropenem, polymyxin-B and sulbactam alone. CLSI breakpoints for interpretation of polymyxin-B MIC results: ≤2 mg/l (intermediate), >2 mg/l (resistant); and meropenem MIC results: ≤ 2 mg/l (susceptible), 4 mg/l (intermediate), and ≥ 8 mg/l (resistant) for *A. baumannii*.

There were no changes in MIC of meropenem with the addition of sulbactam which was fixed at 4 mg/l (threshold concentration of sulbactam), while the MICs of polymyxin-B with the addition of sulbactam in five strains were lower than polymyxin-B alone. Polymyxin-B/meropenem combination reduced the MIC of polymyxin-B and meropenem to lower than their breakpoints (2 mg/l) in 2 of the 11 strains, and the FICI scores were less than 0.5 for 8/11 strains. The addition of 4 mg/l sulbactam to the meropenem/polymyxin-B combination further lowered MIC values to susceptible and intermediate categories in 82% of the isolates. The MIC_50_ values in the triple combination were 2 and 4 mg/l for polymyxin-B and meropenem, respectively. The difference in MIC_50_ between combinations with and without sulbactam was not more than two-fold.

The MPC values of polymyxin-B and meropenem alone or in combination in four of the 11 clinical isolates are shown in Table 4. The MPC values of polymyxin-B alone ranged from 16 to 64 mg/l, and the MPC values of meropenem alone were greater than 128 mg/l. Both polymyxin-B/meropenem and polymyxin-B/meropenem/sulbactam combinations significantly reduced their MPC values against these four isolates to 2 or 4 mg/l.

**Table 4:** Mutant prevention concentrations of meropenem and polymyxin-B alone or in combination with or without sulbactam (fixed at 4 mg/l) against four *A. baumannii* isolates harboring OXA-23 and other serine-β-lactamases.

Clinical isolates	Mutant prevention concentration (mg/L)			
	Monotherapy		Combination therapy	
	meropenem alone	polymyxin-B alone	meropenem/polymyxin-B	meropenem/polymyxin-B/sulbactam
A	>128	32	4/4	4/2/4
E	>128	64	4/4	4/4/4
2	>128	64	4/4	4/4/4
20	>128	16	4/2	4/2/4

MPC, mutant prevention concentration.

### Time-kill kinetics

**Figure 1:**
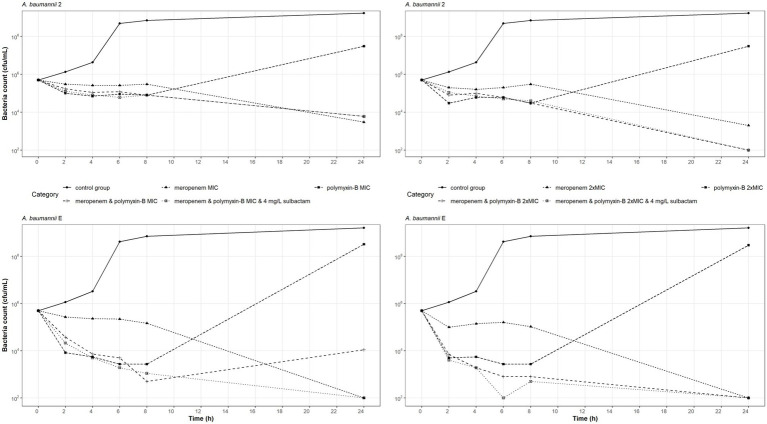
Static-concentration time-kill kinetics of meropenem and polymyxin-B alone and in combination at their respective MIC and 2 × MIC and also as triple combination with 4 mg/l sulbactam against two *A. baumannii* isolates. Monotherapy MICs for meropenem and polymyxin-B were 16 and 16 mg/l for isolate 2, and 128 and 16 mg/l for isolate E, respectively. MICs of meropenem and polymyxin-B were 4 and 4 mg/l for isolate 2, and 4 and < 1 mg/l for isolate E, respectively, in both the double and triple combinations.

Time-kill experiments were conducted on two carbapenem-and polymyxin-resistant *A. baumannii* isolates (2 and E) at the MIC and 2 × MIC of meropenem, and polymyxin-B as monotherapy and also in combination with and without sulbactam (Figure 1). The bacteria that were treated with polymyxin-B alone at MIC or 2 × MIC had a reduction in bacteria burden during the first 8 h but regrew to a density > 10^7^ cfu/ml at 24 h. The two *A. baumannii* isolates may have acquired heteroresistance to polymyxin-B when treated with polymyxin-B alone.

Meropenem monotherapy and meropenem/polymyxin-B combination with or without sulbactam resulted in approximately a 2-log_10_ kill at 24-h, except for meropenem and polymyxin-B at MIC against isolate E (Figure 1 lower left). Meropenem monotherapy MICs against isolates 2 and E were 16 and 128 mg/l, respectively, which were significantly higher than the meropenem MICs in combination therapies (4 and 4 mg/l, respectively). A 2-log_10_ kill at 24-h in the two isolates after meropenem monotherapy is not unexpected.

The MIC values of meropenem and polymyxin-B tested against these two isolates were identical whether 4 mg/l sulbactam was added or not. Sulbactam provided some advantage against *A. baumannii* E tested at MIC of both meropenem and polymyxin-B, resulting in a 24-h bacteria burden at the lower limit of detection. Without sulbactam, the 24-h bacteria burden was 10^4^ cfu/ml. Against *A. baumannii* 2, the additional antimicrobial activities due to sulbactam were marginal.

### Pharmacodynamic analysis of resistant mutant selections

**Figure 2:**
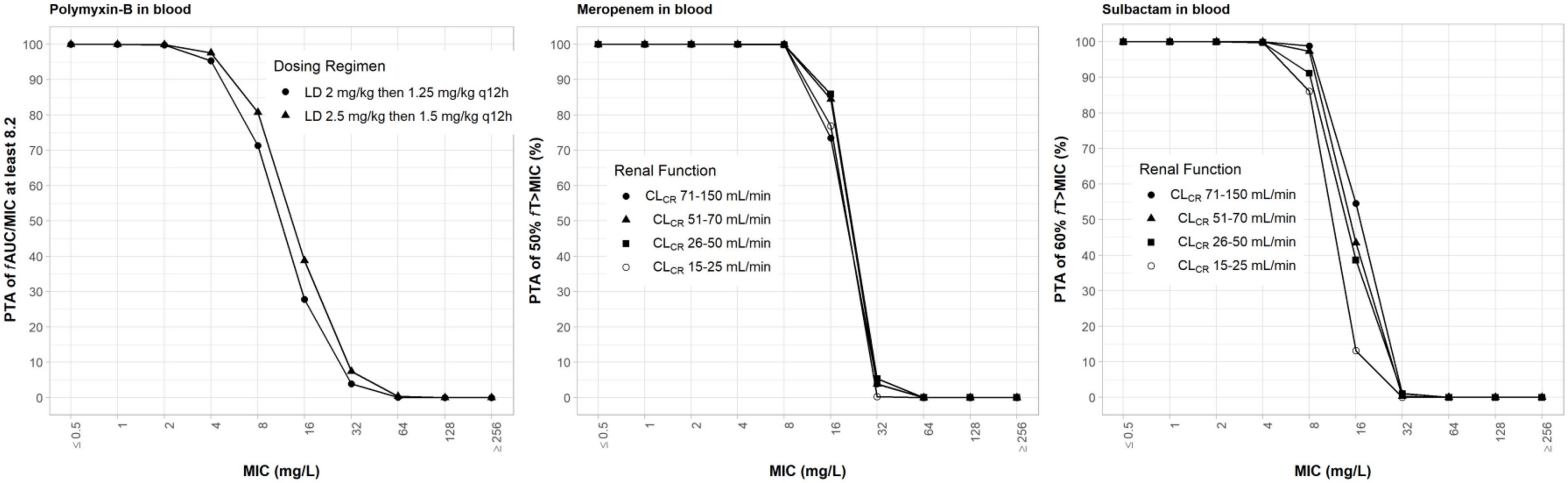
Probability of target attainment of 50% *f*T_>MIC_ and 60% *f*T_>MIC_ for meropenem and sulbactam dosing regimens, respectively, by renal function category and PTA of *f*AUC/MIC of at least 8.2 for polymyxin-B dosing regimens. Probability of target attainment values were computed based on steady-state drug concentrations in the blood. LD, loading dose; CL_CR_, creatinine clearance.

The clinical dosing regimens for the three antibiotics evaluated in the simulations are listed in Table 1. High-dose sulbactam regimens by categories of renal function were selected such that a PTA ≥90% was achieved for 60% *f*T_>MIC_ at the MIC of 4 mg/l (Yokoyama et al., 2014, 2015) in the plasma. Meropenem dosing regimens were based on the recommended maximum total daily dose by renal functions. Both meropenem and sulbactam dosing regimens were selected assuming that both drugs can be administered at the same time. Since polymyxin-B is eliminated by non-renal pathways (Abdelraouf et al., 2012; Manchandani et al., 2016), we evaluated the upper and lower ranges of the recommended regimens without consideration for renal function. The PTA values for the respective PD indices of polymyxin-B, meropenem and sulbactam are shown in Figure 2; ≥90% PTA based on steady-state plasma drug exposure was achieved at 4, 8, and 4 mg/l, respectively.

We evaluated the hypothesis that polymyxin-B/meropenem or polymyxin-B/sulbactam/meropenem would reduce the *f*T_MSW_ and increase *f*T_>MPC_, compared to meropenem or polymyxin-B monotherapy. In the monotherapy scenario, the MPC was greater than the simulated maximum drug concentrations. When *f*T_>MPC_ was 0, *f*T_MSW_ was not determinable. In the co-administration scenarios, these two PD indices were determinable for the four isolates.

The evaluation of these PD indices associated with inhibition of drug resistance is supported for these dosing regimens provided that sufficient PTA is achieved at the MIC in the combination therapy. For both meropenem and polymyxin-B exposures in the blood, ≥90% PTAs were achieved at 8 and 4 mg/l, respectively (Figure 2). For high-dose sulbactam regimens, 100% PTA was achieved for MIC of 4 mg/l. In all isolates (Table 5), mean *f*T_>MPC_ values were over 90%. In two isolates (2 and E), the *f*T_MSW_ were 0%, whereas mean *f*T_MSW_ values were < 10% for the other isolates. Meropenem dosing regimen of 1 g q24h as 4 h infusion in patients with CL_CR_ of >5 to 10 ml/min was relatively flat and resulted in a slightly higher mean *f*T_MSW_ of about 6%.

**Table 5:** Pharmacodynamic parameters *f*T_MSW_ and *f*T_>MPC_ based on MIC and MPC of meropenem in plasma against four *A. baumannii* isolates harboring OXA-23 and other serine-β-lactamases.

**Bacteria isolates**	**Meropenem in double-combination of with polymyxin-B** ^**‡**^		**Meropenem in triple-combination of with polymyxin-B and sulbactam** ^**‡**^	
	*f*T_MSW_	*f*T_>MPC_	*f*T_MSW_	*f*T_>MPC_
*CL_CR_* > 70 to 150 ml/min				
E, 2	0%	98.2 ± 4.8%	0%	98.2 ± 4.8%
A	1.7 ± 4.7%	98.2 ± 4.8%	1.7 ± 4.7%	98.2 ± 4.8%
20	1.5 ± 3.9%	98.2 ± 4.8%	1.7 ± 4.7%	98.2 ± 4.8%
*CL_CR_* > 50 to 70 ml/min				
E, 2	0%	99.5 ± 1.8%	0%	99.5 ± 1.8%
A	0.42 ± 1.8%	99.5 ± 1.8%	0.42 ± 1.8%	99.5 ± 1.8%
20	0.34 ± 1.6%	99.5 ± 1.8%	0.42 ± 1.8%	99.5 ± 1.8%
*CL_CR_* > 25 to 50 ml/min				
E, 2	0%	98.9 ± 3.3%	0%	98.9 ± 3.3%
A	0.96 ± 3.1%	98.9 ± 3.3%	0.96 ± 3.1%	98.9 ± 3.3%
20	0.79 ± 2.6%	98.9 ± 3.3%	0.96 ± 3.1%	98.9 ± 3.3%
*CL_CR_* > 10 to 25 ml/min				
E, 2	0%	99.0 ± 1.8%	0%	99.0 ± 1.8%
A	0.89 ± 1.8%	99.0 ± 1.8%	0.89 ± 1.8%	99.0 ± 1.8%
20	0.67 ± 1.6%	99.0 ± 1.8%	0.89 ± 1.8%	99.0 ± 1.8%
*CL_CR_* > 5 to 10 ml/min				
E, 2	0%	94.0 ± 9.5%	0%	94.0 ± 9.5%
A	5.7 ± 9.0%	94.0 ± 9.5%	5.7 ± 9.0%	94.0 ± 9.5%
20	4.8 ± 7.2%	94.0 ± 9.5%	5.7 ± 9.0%	94.0 ± 9.5%

CL_CR_, creatinine clearance; MIC, minimum inhibitory concentration; MPC, mutant prevention concentration; *f*T_MSW_, fraction of time within 24-h that the drug concentration is within mutant selection window; *f*T_>MPC_, fraction of time within 24-h that the drug concentration is above MPC.

‡See Table 1 for list of dosing regimens by renal function category; simulations were based on assumption of 90% PTA achieved in the plasma using polymyxin-B dosing regimens: loading dose 2.5 mg/kg followed by 1.5 mg/kg q12h at 12 h as 1 h infusion.

We assumed that the protein binding of polymyxin-B was 80%. On this basis, plasma pharmacodynamic parameters *f*T_>MPC_ and *f*T_MSW_ of the four isolates were calculated (Table 6). The MSW was closed in 2/4 isolates for polymyxin-B/meropenem combination, and the MSW was closed for an additional isolate (3/4 isolates) when the triple combination was used. For the majority of the isolates, the mean *f*T_>MPC_ were > 70 and > 60% for the polymyxin-B dosing regimen consisting of a loading dose of 2.5 mg/kg followed by 1.5 mg/kg q12h at 12 h and 2 mg/kg loading dose followed by 1.25 mg/kg q12h at 12 h, respectively. For isolate 20, *f*T_>MPC_ was approximately 90%.

**Table 6:** Pharmacodynamic parameters *f*T_MSW_ and *f*T_>MPC_ based on MIC and MPC of polymyxin-B in plasma against four *A. baumannii* isolates harboring OXA-23 and other serine-β-lactamases.

**Bacteria Isolate**	**Polymyxin-B in double-combination with meropenem** ^**‡**^		**Polymyxin-B in triple-combination with meropenem and sulbactam** ^**‡**^	
	*f*T_MSW_	*f*T_>MPC_	*f*T_MSW_	*f*T_>MPC_
*Loading dose 2.5 mg/kg followed by 1.5 mg/kg q12h at 12 h as 1 h infusion*				
A	23.7 ± 28.8%	70.2 ± 35.5%	0%	93.9 ± 17.7%
E	29.2 ± 34.7%	70.2 ± 35.5%	29.2 ± 34.7%	70.2 ± 35.5%
2	0%	70.2 ± 35.5%	0%	70.2 ± 35.5%
20	0%	93.9 ± 17.7%	0%	93.9 ± 17.7%
*Loading dose 2 mg/kg followed by 1.25 mg/kg q12h at 12 h as 1 h infusion*				
A	29.4 ± 30.0%	60.5 ± 38.0%	0%	89.9 ± 22.8%
E	38.3 ± 36.9%	60.5 ± 38.0%	38.3 ± 36.9%	60.5 ± 38.0%
2	0%	60.5 ± 38.0%	0%	60.5 ± 38.0%
20	0%	89.9 ± 22.8%	0%	89.9 ± 22.8%

MIC, minimum inhibitory concentration; MPC, mutant prevention concentration; *f*T_MSW_, fraction of time within 24-h that the drug concentration is within mutant selection window; *f*T_>MPC_, fraction of time within 24-h that the drug concentration is above MPC.

‡See Table 1 for list of dosing regimens by renal function category; simulations were based on assumption of 90% PTA achieved in the plasma using meropenem/sulbactam dosing regimens of 2 g q8h / 3 g q6h as 3 h infusion.

**Figure 3:**
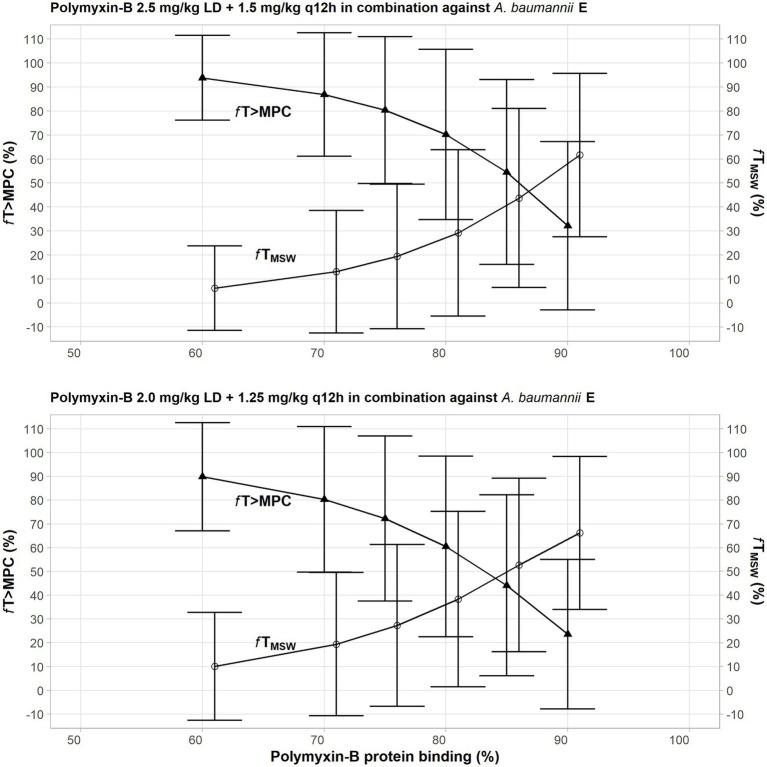
Sensitivity analysis to evaluate effect of variability in polymyxin plasma protein binding on the pharmacodynamic parameters *f*T_MSW_ and *f*T_>MPC_ after polymyxin dosing regimens in combination therapy consisting of 2.5 mg/kg loading dose followed by 1.5 mg/kg q12h at 12 h (top graph) and 2 mg/kg loading dose followed by 1.25 mg/kg q12h at 12 h (bottom graph) against *A. baumannii* E. The models assumed polymyxin MIC of 1 mg/l and MPC of 4 mg/l, whereas meropenem MIC and MPC were both 4 mg/l with or without 4 mg/l sulbactam. In this scenario, the proposed dosing regimens of both meropenem and sulbactam can achieve PTA ≥90%.

Due to a highly variable protein binding of polymyxin-B, a sensitivity analysis was performed to illustrate the effects of variance of protein binding on these two PD parameters (Figure 3). We selected isolate E, because the MSW was not closed in the meropenem/polymyxin-B combination with and without sulbactam. When polymyxin-B plasma protein binding increased from 50 to 90%, the *f*T_MSW_ of polymyxin-B combined with meropenem decreased from over 90% to slightly over 30%, whereas *f*T_>MPC_ increased from <10 to >60% for the 2.5 mg/kg loading dose followed by 1.5 mg/kg q12h dosing regimen. For the 2.0 mg/kg loading dose followed by a 1.25 mg/kg q12h regimen, the *f*T_MSW_ of polymyxin-B combined with meropenem and sulbactam decreased from 90 to <30%, and *f*T_>MPC_ increased from 10 to >60%. The results indicate the sensitivity of the two PD parameters to the availability of free drug concentration of polymyxin-B in the blood.

### Pharmacokinetic and pharmacodynamic analyses of drugs in epithelial lining fluid

**Figure 4:**
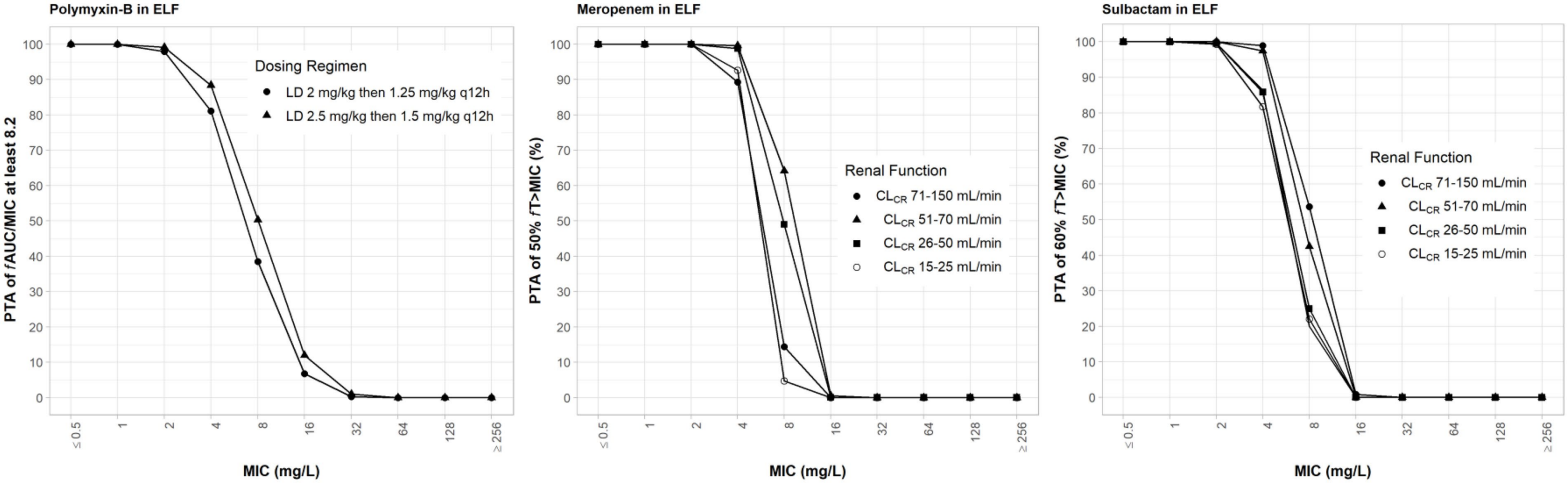
Probability of target attainment of 50% *f*T_>MIC_ and 60% *f*T_>MIC_ for meropenem and sulbactam dosing regimens, respectively, by renal function categoryand PTA of *f*AUC/MIC of at least 8.2 for polymyxin-B dosing regimens. Probability of target attainment values were computed based on steady-state drug concentrations in the epithelial lining fluid (ELF) and their respective ELF penetration. LD, loading dose; CL_CR_, creatinine clearance.

Drug exposures in the ELF were lower than their exposures in the plasma; we assumed 30, 52 and 60% ELF penetration rates for meropenem, sulbactam and polymyxin-B, respectively, after intravenous administration (Lodise et al., 2011; He et al., 2013; Rodvold et al., 2018). For both meropenem and polymyxin-B, ≥80% PTA was achieved at their respective breakpoints (Figure 4). For high-dose sulbactam regimens, ≥80% PTA was achieved for MIC of 4 mg/l in the ELF.

While the *f*T_>MPC_ values for meropenem in the ELF were decreased compared to that in the plasma, the *f*T_MSW_ values were below 50% for isolates whose MSW was not closed (Table 7). For polymyxin-B in combination with meropenem, *f*T_>MPC_ values in the ELF were 27 and 19% for the higher and lower dosing regimens, respectively, against isolates A, E and 2 (Table 8). These values were 65 and 55%, respectively, against isolate 20. The addition of sulbactam improved the *f*T_>MPC_ and closed the MSW against isolate A.

**Table 7:** Pharmacodynamic parameters *f*T_MSW_ and *f*T_>MPC_ based on MIC and MPC of meropenem in the epithelial lining fluid against four *A. baumannii* isolates harboring OXA-23 and other serine-β-lactamases.

**Bacteria Isolate**	**Meropenem in double-combination with polymyxin-B** ^**‡**^		**Meropenem in triple-combination with polymyxin-B and sulbactam** ^**‡**^	
	*f*T_MSW_	*f*T_>MPC_	*f*T_MSW_	*f*T_>MPC_
*CL_CR_ > 70 to 150 ml/min*				
E, 2	0%	74.2 ± 18.1%	0%	74.2 ± 18.1%
A	24.7 ± 16.8%	74.2 ± 18.1%	24.7 ± 16.8%	74.2 ± 18.1%
20	19.5 ± 11.8%	74.2 ± 18.1%	24.7 ± 16.8%	74.2 ± 18.1%
*CL_CR_ > 50 to 70 ml/min*				
E, 2	0%	90.0 ± 11.9%	0%	90.0 ± 11.9%
A	9.7 ± 11.6%	90.0 ± 11.9%	9.7 ± 11.6%	90.0 ± 11.9%
20	8.4 ± 9.5%	90.0 ± 11.9%	9.7 ± 11.6%	90.0 ± 11.9%
*CL_CR_* > 25 to 50 ml/min				
E, 2	0%	84.7 ± 14.3%	0%	84.7 ± 14.3%
A	14.6 ± 13.4%	84.7 ± 14.3%	14.6 ± 13.4%	84.7 ± 14.3%
20	11.9 ± 10.1%	84.7 ± 14.3%	14.6 ± 13.4%	84.7 ± 14.3%
*CL_CR_* > 10 to 25 ml/min				
E, 2	0%	77.2 ± 16.1%	0%	77.2 ± 16.1%
A	22.1 ± 15.9%	77.2 ± 16.1%	22.1 ± 15.9%	77.2 ± 16.1%
20	19.5 ± 13.3%	77.2 ± 16.1%	22.1 ± 15.9%	77.2 ± 16.1%
*CL_CR_ > 5 to 10 ml/min*				
E, 2	0%	48.5 ± 13.3%	0%	48.5 ± 13.3%
A	47.5 ± 11.2%	48.5 ± 13.3%	47.5 ± 11.2%	48.5 ± 13.3%
20	33.7 ± 8.1%	48.5 ± 13.3%	47.5 ± 11.2%	48.5 ± 13.3%

CL_CR_, creatinine clearance; MIC, minimum inhibitory concentration; MPC, mutant prevention concentration; *f*T_MSW_, fraction of time within 24-h that the drug concentration is within mutant selection window; *f*T_>MPC_, fraction of time within 24-h that the drug concentration is above MPC.

‡See Table 1 for list of dosing regimens by renal function category; simulations were based on assumption of 90% PTA achieved in the plasma using meropenem/sulbactam dosing regimens of 2 g q8h / 3 g q6h as 3 h infusion.

**Table 8:** Pharmacodynamic parameters *f*T_MSW_ and *f*T_>MPC_ based on MIC and MPC of polymyxin-B in the epithelial lining fluid against four *A. baumannii* isolates harboring OXA-23 and other serine-β-lactamases.

**Bacteria Isolate**	**Polymyxin-B in double-combination with meropenem** ^**‡**^		**Polymyxin-B in triple-combination with meropenem and sulbactam** ^**‡**^	
	*f*T_MSW_	*f*T_>MPC_	*f*T_MSW_	*f*T_>MPC_
*Loading dose 2.5 mg/kg followed by 1.5 mg/kg q12h at 12 h as 1 h infusion*				
A	37.6 ± 28.8%	27.1 ± 33.1%	0%	64.7 ± 32.8%
E	64.7 ± 32.8%	27.1 ± 33.1%	64.7 ± 32.8%	27.1 ± 33.1%
2	0%	27.1 ± 33.1%	0%	27.1 ± 33.1%
20	0%	64.7 ± 32.8%	0%	64.7 ± 32.8%
*Loading dose 2 mg/kg followed by 1.25 mg/kg q12h at 12 h as 1 h infusion*				
A	35.3 ± 28.4%	19.3 ± 28.9%	0%	54.6 ± 38.6%
E	67.6 ± 31.6%	19.3 ± 28.9%	67.6 ± 31.6%	19.3 ± 28.9%
2	0%	19.3 ± 28.9%	0%	19.3 ± 28.9%
20	0%	54.6 ± 38.6%	0%	54.6 ± 38.6%

MIC, minimum inhibitory concentration; MPC, mutant prevention concentration; *f*T_MSW_, fraction of time within 24-h that the drug concentration is within mutant selection window; *f*T_>MPC_, fraction of time within 24-h that the drug concentration is above MPC.

‡See Table 1 for list of dosing regimens by renal function category; simulations were based on assumption of 90% PTA achieved in the plasma using meropenem/sulbactam dosing regimens of 2 g q8h / 3 g q6h as 3 h infusion.

## Discussion

OXA-23-producing CRAB is widespread in many parts of the world (Al Atrouni et al., 2016). Polymyxin-B has re-emerged as the antibiotic of “last resort” against CRAB infections, but its use as monotherapy in the clinic is limited due to the rapid emergence of resistant strains (Qureshi et al., 2015). The current study evaluated PD parameters that are associated with the selection for the resistant mutant of *A. baumannii* harboring OXA-23 when administered either meropenem or polymyxin-B alone and in combination with or without sulbactam.

We observed that there was no reduction in meropenem MIC in all isolates tested when 4 mg/l sulbactam was added. Carbapenemases in these isolates were not inhibited by sulbactam, since sulbactam did not improve meropenem activities. OXA-23 is not inhibited by sulbactam and can confer sulbactam resistance in *A. baumannii* (Yang et al., 2019). Antimicrobials of different mechanisms of action are more likely to exert additional benefits such as sulbactam and polymyxin-B, even though the reduced polymyxin-B MIC in these isolates did not reach the clinical breakpoint when sulbactam was added. The sulbactam/meropenem/polymyxin-B combination lowered MIC values to susceptible/intermediate criteria for meropenem and polymyxin-B, respectively, in 5/11 of the isolates, while the combination of meropenem/polymyxin-B could only reduce their MIC to their breakpoints in 2/11 of the isolates. Sulbactam could provide additional benefits to meropenem/polymyxin-B combination and expand their application in the clinic, as we have recently shown that sulbactam disrupted metabolomic pathways involved in peptidoglycan synthesis within 15 min. of treatment (Zhu et al., 2022c). The ability of sulbactam to improve the performance of meropenem and polymyxin-B may be understated because only a single and relatively low concentration of sulbactam was used in the *in vitro* experiments. Sulbactam has intrinsic antibacterial activity against *A. baumannii* through the disruption of bacterial cell wall synthesis (Corbella et al., 1998; Lin et al., 2014). Studies showed that combining sulbactam with other antibacterial agents could enhance their bacterial killing effects (Qureshi et al., 2015; Lenhard et al., 2017; Kengkla et al., 2018; Menegucci et al., 2019).

Polymyxin-B, as a cationic antimicrobial peptide, is attracted to the negatively charged lipopolysaccharides (LPSs) of bacteria. Bacteria protect themselves by altering the LPS structure through a cationic substitution of the phosphate groups by L-Ara4N or the addition of phosphoethanolamine, which decreases the overall negative charge (Olaitan et al., 2014). Patients who received polymyxins for the treatment of polymyxins-susceptible and CRAB infection could expect polymyxin-B heteroresistance (Lean et al., 2014; Qureshi et al., 2015). In 2020, CLSI adjusted the polymyxin interpretation to remove the susceptible category because of the number of polymyxin treatment failures and the development of resistance during polymyxin monotherapies (Satlin et al., 2020). In the time-kill experiment, we observed that polymyxin-B alone at MIC and 2 × MIC resulted in bacteria regrowth at 24 h, whereas the addition of meropenem with or without sulbactam resulted in no bacteria regrowth at 24 h. We note that the polymyxin-B concentrations used in the time-kill experiment for MIC and 2 × MIC in the monotherapy setting are unlikely to be achieved in the clinical setting *via* intravenous dosing. In order to avoid polymyxin-B heteroresistance, combination therapy is recommended (Bergen et al., 2015). Given that hospital infections are treated empirically, one should consider the small spectrum of activity of sulbactam and the emergence of resistance if polymyxin-B is used. It is important to find an optimal way of using polymyxins in order to reduce the risk of developing heteroresistance (Lenhard et al., 2017). Qureshi and colleagues indicated that the treatment regimen for colistin-resistant *A. baumannii* infection consisting of a carbapenem, colistin methanesulfonate, and ampicillin/sulbactam was associated with the lowest mortality rate in patients with mostly ventilator-associated pneumonia (Qureshi et al., 2015). Lenhard and colleagues simulated a dynamic time-kill of meropenem/sulbactam/polymyxin-B combination regimen against a pan-resistant *A. baumannii* isolate collected from a critically ill patient in a hollow fiber infection model and showed that the triple combination eradicated the pathogen in 96 h, whereas monotherapies and double combination resulted in regrowth (Lenhard et al., 2017).

The PTA of polymyxin-B dosing regimens in both plasma and ELF is an indicator of treatment efficacy against the pathogen based on drug exposures in the blood and the lung (Zhu et al., 2022b). Even with ≥80% PTA for polymyxin-B exposure in the ELF, *f*T_>MPC_ in the ELF after polymyxin-B intravenous administration can be limited to around 20%. Unless the MSW is closed by combination therapy, the limited free drug in the ELF may provide ample opportunities for the resistant mutant pathogens to proliferate. Aerosolized polymyxin-B is being used in the clinic (Pereira et al., 2007), and could increase drug availability to the lung. The international consensus guidelines for the optimal use of polymyxins recommended (weakly) that inhaled polymyxins may be used adjunctively with intravenous polymyxins to treat HAP and VAP (Tsuji et al., 2019). In a mouse lung infection model, aerosolized polymyxin-B resulted in a maximum drug concentration (C_max_) of >100 mg/l in the ELF after 4.12 mg/kg and 8.24 mg/kg doses; polymyxin-B aerosols significantly reduced lung inflammation in the animal (Lin et al., 2017). The choice of antibiotic in the combination is important; the ELF penetration should also be considered when treating lung-related infections due to CRAB.

A word of caution is warranted. The benefit–risk ratio of the triple antibiotic combination should be weighed against their potential toxicities and alterations on the microbiome. Unlike efficacy, synergistic adverse effects due to combination antibiotics are not well documented. Though these antibiotics are intended for parenteral administration, the intestinal microbiota can potentially be disrupted, resulting in a loss of resistance to colonization by pathogenic bacteria. In this way, nosocomial infections resistant to antibiotics can derive from gastrointestinal colonization. However, there are survival benefits of combination antibiotic therapy in the most severely ill patients (Kumar et al., 2010). A judgment call should be made on whether to treat patients with additional antibiotics or to consider an additional alternate route of administration such as inhaled antibiotics, given the trade-off between marginal benefits from the triple antibiotics and their potential adverse effects.

This *in vitro* and simulation study shows the importance of measuring drug exposure at the sites of infection. Antimicrobial combinations can restrict the emergence and spread of antibiotic resistance following drug exposure. An intravenous polymyxin-B combination with an adjunctive inhaled polymyxin is promising in combatting pneumonia caused by CRAB. However, additional studies will be warranted including a clinical confirmation.

## Data availability statement

The original contributions presented in the study are included in the article/Supplementary material, further inquiries can be directed to the corresponding authors.

## Ethics statement

This study was carried out in accordance with the guidance for the collection and testing of clinical samples. The protocol was approved by the Medical Ethics Committee of the Affiliated Hospital of Qingdao University (Approval No.: QDFY20180512).

## Author contributions

JZ, SD, YaL, HW, YuL, SZ, KF, XT, CO, PZ, ZL, MY, SS, YZ contributed to the design of the study, acquisition, or analysis of data, drafted or revised the article for intellectual content, and approved the final version. All authors contributed to the article and approved the submitted version.

## Funding

This work was supported by grants from the Foundation of the Affiliated Hospital of Qingdao University (2019 + X), Shandong Provincial Natural Science Foundation (ZR2019BC025).

## Conflict of interest

The authors declare that the research was conducted in the absence of any commercial or financial relationships that could be construed as a potential conflict of interest.

## Publisher’s note

All claims expressed in this article are solely those of the authors and do not necessarily represent those of their affiliated organizations, or those of the publisher, the editors and the reviewers. Any product that may be evaluated in this article, or claim that may be made by its manufacturer, is not guaranteed or endorsed by the publisher.
