# Supplementary material for: The combination effect of meropenem/sulbactam/polymyxin-B on the pharmacodynamic parameters for mutant selection windows against carbapenem-resistant Acinetobacter baumannii
Source: Front Microbiol. 2022 Nov 22;13:1024702. doi: 10.3389/fmicb.2022.1024702 (PMC9723340; doi:10.3389/fmicb.2022.1024702)
Supplement: Supplementary file 2 [file Data_Sheet_2.docx]

**Supplemental Material for:**

**The combination effect of meropenem/sulbactam/polymyxin-B on the pharmacodynamic parameters for mutant selection windows against carbapenem-resistant *Acinetobacter baumannii***

**Description of population pharmacokinetic models**

A summary of population pharmacokinetic model parameters is listed in Table S1. For meropenem, a two-compartment ([Ehmann et al., 2019](#_ENREF_1)) was utilized wherein drug clearance (CL, L/h) was dependent on CL_CR_. Central volume (V_C_, L) was influenced by body weight whereas albumin (ALB, g/dL) influenced peripheral volume (V_P_, L). These model parameters were consistent with reported values in critically ill patients ([Isla et al., 2008](#_ENREF_2);[Usman et al., 2017](#_ENREF_6);[Minichmayr et al., 2018](#_ENREF_3)).

The model for sulbactam was also a 2-compartment model based on that reported for community-acquired pneumonia patients ([Soto et al., 2014](#_ENREF_5)). Its clearance is influenced by creatinine clearance whereas body weight is a covariate of the peripheral volume of distribution.

The model for polymyxin-B in critically ill patients was a 2-compartment model ([Sandri et al., 2013](#_ENREF_4)) parameterized on weight-scaled parameters. Individual parameters were obtained by multiplying weight-scaled parameters with the virtual patient’s weight.

Table S1: Summary of population pharmacokinetic parameters of antibiotics used in the simulation

| **Drug** | **Meropenem** | **Sulbactam** | **Polymyxin-B** |
| --- | --- | --- | --- |
| Route of administration | intravenous | intravenous | Intravenous |
| Reference | ([Ehmann et al., 2019](#_ENREF_1)) | ([Soto et al., 2014](#_ENREF_5)) | ([Sandri et al., 2013](#_ENREF_4)) |
| Population | 48 critically ill Caucasian patients | 47 Japanese patients with community-acquired  pneumonia | 24 critically ill Brazil patients |
| CLcr (mL/min) | Not available | 71.0 (34.6-176) | 33 (10–143) |
| Age (yrs) | Not available | 28-85 | 21-87 |
| Infusion time (h) | 0.5 h, 3 h, 24 h | 0.5 h infusion | 1 – 4 h |
| Dosing regimen | 1000 – 2000 mg q8h or q12h;  500 mg as 0.5 h infusion followed by 3000 mg q24h;  1000 mg as 0.5 h infusion followed by 6000 mg q24h continuous infusion | 3g | 0.45–3.38 mg/kg/day q12h or 24h |
| No. compartment | 2 | 2 | 2 |
| Pop PK parameters | If CLcr < 154 mL/min,  CL = 9.25 (1+ 0.009977(CLcr-80.8))  If CLcr ≥ 154 mL/min,  CL (L/h) = 9.25 (1+ 0.009977(154-80.8))  CV% CL = 27.1  V_C_ (L) = 7.89* (WT/70)^0.945^  CV% V_C_ = 31.5  V_P_ (L) = 16.1*(1-0.202*(ALB-2.79))  CV% V_P_ = 16.9  Q (L/h) = 28.4 | CL (L/h) = 10.4 × (CLcr/71)^0.701^  CV% CL = 15.2  V_C_ (L) = 10.2  Q (L/h) = 4.58  V_P_ (L) = 4.04 (WT/51)  CV% V_P_ = 14.8 | CL (L/h/kg) = 0.0276  CV% CL = 32.4%  Q (L/h/kg) = 0.146  CV% Q = 50.4%  V_C_ (L/kg) = 0.0939  CV% V_C_ = 73.3%  V_P_ (L/kg) = 0.330  CV% V_P_ = 70.1% |
| Covariates | CLcr, WT, ALB | CLcr, WT | WT |

ALB, albumin; CLcr, creatinine clearance; WT, body weight; CL, drug clearance; Q, intercompartmental clearance; V_C_, central volume; V_P_, peripheral volume

**References**

Ehmann, L., Zoller, M., Minichmayr, I.K., Scharf, C., Huisinga, W., Zander, J., and Kloft, C. (2019). Development of a dosing algorithm for meropenem in critically ill patients based on a population pharmacokinetic/pharmacodynamic analysis. *Int J Antimicrob Agents* 54**,** 309-317.

Isla, A., Rodriguez-Gascon, A., Troconiz, I.F., Bueno, L., Solinis, M.A., Maynar, J., Sanchez-Izquierdo, J.A., and Pedraz, J.L. (2008). Population pharmacokinetics of meropenem in critically ill patients undergoing continuous renal replacement therapy. *Clin Pharmacokinet* 47**,** 173-180.

Minichmayr, I.K., Roberts, J.A., Frey, O.R., Roehr, A.C., Kloft, C., and Brinkmann, A. (2018). Development of a dosing nomogram for continuous-infusion meropenem in critically ill patients based on a validated population pharmacokinetic model. *J Antimicrob Chemother* 73**,** 1330-1339.

Sandri, A.M., Landersdorfer, C.B., Jacob, J., Boniatti, M.M., Dalarosa, M.G., Falci, D.R., Behle, T.F., Saitovitch, D., Wang, J., Forrest, A., Nation, R.L., Zavascki, A.P., and Li, J. (2013). Pharmacokinetics of polymyxin B in patients on continuous venovenous haemodialysis. *J Antimicrob Chemother* 68**,** 674-677.

Soto, E., Shoji, S., Muto, C., Tomono, Y., and Marshall, S. (2014). Population pharmacokinetics of ampicillin and sulbactam in patients with community-acquired pneumonia: evaluation of the impact of renal impairment. *Br J Clin Pharmacol* 77**,** 509-521.

Usman, M., Frey, O.R., and Hempel, G. (2017). Population pharmacokinetics of meropenem in elderly patients: dosing simulations based on renal function. *Eur J Clin Pharmacol* 73**,** 333-342.
